# Genomic Changes in an Attenuated ZB Strain of Foot-and-Mouth Disease Virus Serotype Asia1 and Comparison with Its Virulent Parental Strain

**DOI:** 10.1155/2014/978609

**Published:** 2014-10-19

**Authors:** Aiguo Xin, Mingwang Zhu, Zhenqi Peng, Qi Hu, Chenhong Shi, Defang Liao, Jihua Wang, Huachun Li

**Affiliations:** ^1^Yunnan Tropical and Subtropical Animal Virus Disease Laboratory, Yunnan Animal Science and Veterinary Institute, Kunming 650224, China; ^2^Yunnan Provincial Research Center for Veterinary Biological Products, Baoshan 678000, China

## Abstract

The molecular basis of attenuation of foot-and-mouth disease virus (FMDV) serotype Asia1 ZB strain remains unknown. To understand the genetic changes of attenuation, we compared the entire genomes of three different rabbit-passaged attenuated ZB strains (ZB/CHA/58(att), ZBRF168, and ZBRF188) and their virulent parental strains (ZBCF22 and YNBS/58). The results showed that attenuation may be brought about by 28 common amino acid substitutions in the coding region, with one nucleotide point mutation in the 5′-untranslated region (5′-UTR) and another one in the 3′-UTR. In addition, a total of 21 nucleotides silent mutations had been found after attenuation. These substitutions, alone or in combination, may be responsible for the attenuated phenotype of the ZB strain in cattle. This will contribute to elucidation of attenuating molecular basis of the FMDV ZB strain.

## 1. Introduction

Foot-and-mouth disease (FMD) is a highly infectious disease of cloven-hoofed animals. The causative agent, foot-and-mouth disease virus (FMDV), is a member of the genus* Aphthovirus* within the family* Picornaviridae* and has seven serotypes: O, A, C, Asia1, and Southern African Territories (SAT) 1, SAT2, and SAT3 [1, 2]. Infection or vaccination with one serotype of FMDV does not protect against other serotypes [3]. The FMDV genome includes a single open reading frame (ORF) flanked by 5′ and 3′ untranslated regions, 5′-UTR and 3′-UTR. Upon infection, the viral RNA is translated into a single polyprotein that is concurrently processed by three virus-encoded proteinases, leader (L^pro^), 2A, and 3C^pro^ into precursors and consequent mature structural (VP1, VP2, VP3, and VP4) and nonstructural (L^pro^, 2A, 2B, 2C, 3A, 3B, 3C^pro^, and 3D^pol^) proteins [4, 5].

In the late 1950s, a FMD-like disease occurred in Baoshan county, Yunnan province, China. A virus related to the outbreak was isolated and named as the FMDV ZB (Zhongguo Baoshan) strain. Subsequently, a live attenuated vaccine was developed by serial passaging wild-type virulent ZB strain in suckling rabbits for more than 100 passages, in order to prevent FMD Asia1 outbreaks on the border between China and Myanmar from 1960s to 1990s. A review of the passaging history of the virulent ZB strain attenuation process revealed that the virus after the 60th passage still caused clinical FMD but did not cause visible clinical symptoms in cattle after 109th passage. The virus passaged to the 114th passage was therefore used as a live vaccine after its safety and efficacy were confirmed.

However, the criteria for the selection of the attenuated strain were highly empirical, and little is known about the molecular mechanisms causing attenuation. The complete genome sequence of the attenuated strain and its virulence determinants must be clear for quality control of the vaccine. Although the entire genomes of a cell-culture rabbit-attenuated ZB/CHA/58(att) strain and an inactivated vaccine YNBS/58 strain deriving from the same origin of ZB strain [6] had been previously compared, the virulence determinants of the ZB strain have not been elucidated. A total of 25 amino acid substitutions were observed between strains ZB/CAH/58(att) and YNBS/58 [7]. Therefore, we compared the entire genomes of three different rabbit-passaged attenuated ZB strains (ZB/CHA/58(att), ZBRF168, and ZBRF188) and their virulent parental viruses (ZBCF22 and YNBS/58), in order to identify genomic changes that occurred during the attenuation process of the ZB strain.

## 2. Materials and Methods

### 2.1. Viruses

Virulent FMDV Asia1 ZBCF22 strain was passaged 22 times in cattle via needle inoculation and then passaged on tongue epithelium in Baoshan County, China. It was maintained as a challenge virus strain at Yunnan Provincial Research Center for Veterinary Biological Products. The attenuated ZBRF168 and ZBRF188 strains were derived from virulent parental ZBCF22 strain via consecutive passage for 168 or 188 times in suckling rabbits following the established protocol [8] with modifications. Briefly, a 5-day-old suckling rabbit was subcutaneously inoculated with the viral agent. The rabbits showed clinical signs of short breath and leg paralysis 16–20 hrs after inoculation and died between 18 and 28 hrs. The carcasses of dead rabbits were harvested and homogenized in PBS buffer at 1/10 (w/v) followed by clarification by centrifugation at 1000 g for 30 min. The supernatant containing the viral agent was then used as inoculums for the next passage. ZB/CHA/58(att) strain is a cell-adapted rabbit-attenuated strain that was passaged 187 times in suckling rabbits and adapted to BHK-21 cells. These attenuated strains are stored at the Yunnan Animal Science and Veterinary Institute.

### 2.2. FMDV RNA Extraction and Reverse Transcription Polymerase Chain Reaction (RT-PCR)

FMDV RNAs were extracted using the RNAiso Plus (TaKaRa Biotechnology Co. Ltd., Dalian, China) and used immediately for cDNA synthesis. Synthesis of cDNA was carried out using 6 random primers and Superscript II reverse transcriptase (Invitrogen, USA). Six cDNA fragments covering the entire FMDV genome were amplified by PCR using six primer sets (Table 1). PCR amplifications with Pyrobest* Pfu *polymerase (TaKaRa Biotechnology Co. Ltd., Dalian, China) were performed according to the manufacturer's protocol. For PCR amplification, PCR reaction conditions were as follows: 1 cycle predenaturation for 5 min at 95°C, 30 cycles for amplification at 94°C for 30 sec, 58°C (for F1 fragment) or 52°C (for F2, F3, F4, F5, and F6 fragment) for 30 sec, 72°C for 1 min (for F1) or 3 min (for F2, F3, F4, F5, and F6), and 1 cycle for final extension at 72°C for 10 min. The PCR products were purified from agarose gel electrophoresis and sequenced directly using a ABI-PRIS MTM 377XL DNA Sequencer at two companies, Sangon Biotech (Shanghai, China) and Taihe Biotech (Beijing, China). The genomic sequences were sequenced twice at each of the companies.

### 2.3. Sequence Analysis

FMDV reference sequences were acquired from the GenBank database of the National Center for Biotechnology Information (http://www.ncbi.nlm.nih.gov/). The sequence data were assembled using the program Assemble (Vector NTI 8.0 suite, InforMax, North Bethesda, MD, USA). Multiple sequence alignments were performed using a ClustalX multiple sequence alignment program, version 1.83 [9]. A phylogenetic tree was constructed by the neighbor-joining method with the Kimura 2-parameter nucleotide substitution method using 1000 bootstrap replicates in the MEGA version 3.1 [10]. RNA secondary structure was extrapolated using the RNA structure 4.6 software.

## 3. Results and Discussion

### 3.1. Genomic Organization of ZB Strains and Phylogenetic Analysis

The genome sequences of the virulent ZBCF22 and the rabbit-attenuated strains (ZBRF168 and ZBRF188) were determined to be 8,164 nucleotides (nt) in length (excluding the poly-C and poly-A tracts) and contain an ORF of 6,990 nt that encodes a polyprotein of 2,329 amino acids, which is consistent with the previously reported ZB/CHA/58(att) genome [7]. The ORFs of these strains were shown to be flanked by a 5′-UTR of 1,081 nt and a 3′-UTR of 93 nt.

**Figure 1:**
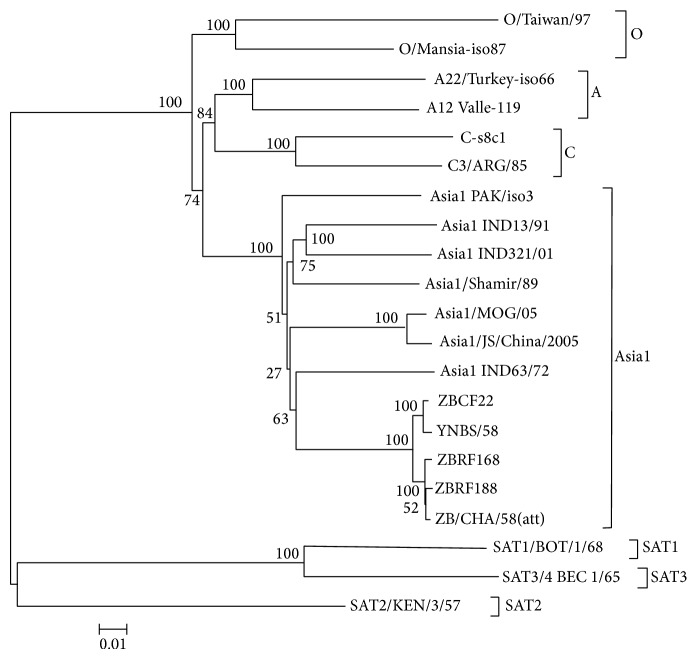
Phylogenetic analysis of the FMDV genome sequences. Phylogenetic tree was constructed by the neighbor-joining method by using MEGA 3.1, and bootstrap values were determined by 1,000 replicates. The GenBank accession numbers of 16 reference strains are listed as follows. O Taiwan/97(AY593835), O/Manisa-iso87(AY593823), A12 Valle strain-119(AY593752), A22 Turkey/65-iso66(AY593765), C1/C-s8cl(AJ133357), C3/Arg/85(AJ007347), SAT1 BOT/1/68(AY593845), SAT2/KEN/57(AY251473), SAT3/4 BEC 1/65(AY593853), Asia1 PAK/iso3(AY593795), Asia1 IND 13/91(DQ989312), Asia1 IND321/01(AY687333), Asia1/Shamir/89(JF739177), Asia1/MOG/05(EF614458), Asia1/JS/CHA/05(EF149009), Asia1/IND 63/72(AY304994), YNBS/58(AY390432), and ZB/CHA/58(att)(DQ533483).

A neighboring-joining (NJ) tree construct was based on the sequence alignment of 21 selected genomes, which were distinctly divided into seven serotypes of FMDV and the ZB strains were tightly clustered in the Asia1 serotype (Figure 1). The results demonstrate that ZB strains belong to the FMDV serotype Asia1 from the perspective of comparative genomics.

### 3.2. Comparison of the Untranslated Region Sequences of Different FMDV Strains

**Figure 2:**
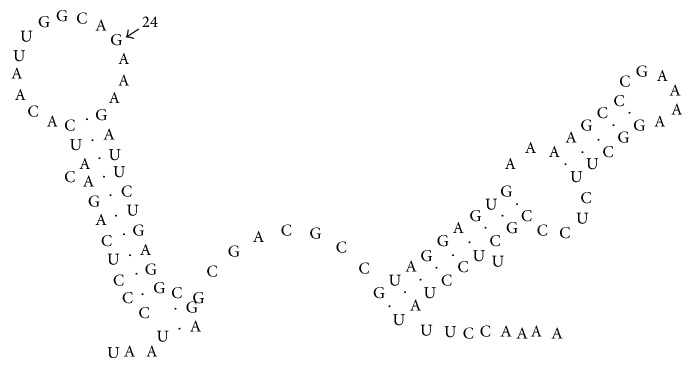
The 3′-UTR RNA secondary structure of FMDV ZBCF22 strain.

The nucleotide sequences were compared for the three attenuated ZB strains, the two virulent strains, and other FMDV Asia1 reference strains. Observed differences in the 5′-UTR between the virulent and attenuated ZB strains were limited, with only one common nucleotide mutated in the internal ribosome entry site (IRES) region (C573-G) (Table 2). The critical role of the IRES element in mediating efficient translation of the viral RNA suggests that this mutation may be involved in the attenuated phenotypes of the ZB strains, presumably through a disruption of RNA secondary structure of IRES. It has been reported that FMD virus after 100 passages in BHK-21 cells carried two point mutations in the IRES and showed increased virulence in cells [11]. In contrast, previous studies have shown that nucleotides changes in the UTR between virulent and attenuated FMDV strains (i.e., strains O1 Campos and C3 Resende) may not be the key determinants of egg-adapted attenuated FMDVs [12]. The FMDV 3′-UTR was predicted to fold into two well-defined stem-loop (SL) structures, highly conserved among isolates and essential for viral infection and IRES activity [13]. Of the two, the first stem-loop (SL1) is to be dispensable for infectivity acting as a replication enhancer [14]. In the genomes of ZBCF22 and ZB/CHA/58(att) strains, guanine was located at the 24 position of SL1 in 3′-UTR (Figure 2), but guanine was replaced by adenine (Table 2) in the genomes of the ZBRF168 and ZBRF188 strains. We conclude that this noncommon mutation in the 3′-UTR is unlikely to be involved in the process of the attenuation.

### 3.3. Comparison of the Protein Coding Regions of FMDVs

Comparison of the protein coding regions between the virulent and attenuated ZB strains revealed no deletion/insertion mutations. A total of 33 amino acid substitutions were observed scattered across nine proteins (L^pro^, VP2, VP1, 2A, 2B, 2C, 3A, 3C^pro^, and 3D^pol^). Five of them (VP2: D133-G, VP1: P146-F, G155-R, 2A: K8-E, and 3A: A51-G) only existed in the rabbit-attenuated ZB/CHA/58(att) strain, whereas 28 common amino acid changes were found during the process of attenuation of the ZB strains. No amino acid changes in the VP4, VP3, and 3B protein had been found after attenuation (Table 3). In addition, a total of 21 characteristic nucleotide substitutions, all of which produce silent mutations, were found scattered across ten proteins (L^pro^, VP4, VP2, VP3, VP1, 2B, 2C, 3A, 3B, and 3D^pol^) (Table 4).

In protease 3C^pro^ of the ZB strains, a common substitution V74-I was observed during the attenuation process, which was identical to the other FMDV Asia1 reference strains (Table 3). Residue V74-I substitution might not be essential for attenuation since both amino acids belong to the same hydrophobic group, although FMDV 3C^pro^ is critical for viral pathogenesis and plays vital roles in both the processing of the polyprotein precursor and RNA replication [15, 16]. In fact, proteases play an essential role in viral polyprotein processing and have been shown to be important virulence determinants in many pathogens [17–19]. In protease L^pro^ of ZB strains, three common amino acid changes (N2-D, M143-L, and E147-G) have been found. Two of them (N2-D and E147-G) changed the charges of the amino acids and thus may likely contribute to the attenuated phenotype of ZB strains. Residue N2-D substitution was located at the inter-AUG region of FMDV, which has been associated in the attenuation of FMDV A24 strain [20]. FMDV L^pro^ was shown to be a virulence determinant based on experiments with L^pro^ lacking virus, which is highly attenuated in cattle and pig [21, 22] and not required for viral replication [16]. L^pro^ plays a central role in pathogenesis through regulation affecting the host innate immune response [23–25]. Residue L143 of the FMDV L^pro^ is a determinant of cleavage specificity, but a hydrophobic residue (M/L) substitution in the L^pro^ may not be the key virulence determinant for the ZB strain attenuation [26].

In the conserved RNA-dependent RNA polymerase 3D^pol^, three common amino acid changes (R84-H, V158-A, and H378-Q) were found after attenuation by passaging* in vivo*. However, four picornaviral polymerase peptide motifs KDELR, PSG, YGGD, and FLKR [27, 28] were conserved among the virulent and attenuated ZB strains, and three hypervariable and hydrophobic antigenic regions (aa 1 to 12, 64 to 76, and 143 to 145) were also stable in all ZB strains [29, 30]. Furthermore, it has been recently reported that the R84-H mutant is a high fidelity variant and does not correlate with virus attenuation [31]. We suspect that residues R84-H and V158-A substitutions are not the key determinants of attenuation, but that the H378-Q mutation (a basic amino acid changed to an amide amino acid) may be related to the attenuation of ZB strains in cattle.

In the structural protein P1-2A region of the ZB strains, no substitution was observed in the VP4 or VP3 protein after the attenuation process. It was previously reported that residue R56-H substitution in the VP3 of O1Campos strain could lead to thermostability and induced typical clinical signs of FMD [32]. We found that a common substitution (V184-L) in the VP2 protein of the ZB strains located at the previously identified T-cell epitopes (aa 179 to 187) [33]. FMDV VP2 associates mainly with virion structural stability and maturation [34]. Therefore, considering that valine and leucine residue share the same amino acid properties, we assume that substitution V184-L may not play a critical role in the attenuation of the ZB strains.

**Figure 3:**
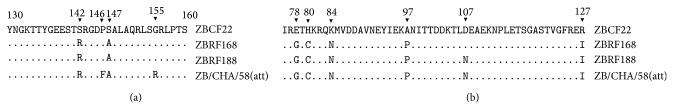
Comparison of amino acid differences between the ZB virulent and attenuated strains: (a) VP1 antigen site B (aa130-160) and (b) 3A (aa76-127).

The highest mutation rate was found in the VP1 protein with eight amino acid substitutions during the attenuation process of the ZB strains (Table 3). Four of them (A4-T, P45-L, A81-V, and S147-A) included amino acids with similar nature, while two others (A21-E and S142-R) resulted in a change of amino acids charge. It was previously proposed that changes of viral surface may play an important role in attenuation process. Therefore, VP1 is likely to be the protein for attenuating mutations. Indeed, a recent study demonstrated that the capsid proteins of the O1Kaubeuren B64 strain were responsible for its attenuation in cattle [35]. Moreover, the adaptive replacement of L147-P in VP1 of the guinea pig-adapted C-S8c1 strain abolished growth of the virus in different established cell lines and modified its antigenicity [36]. Additionally, substitution of D143-A in the G-H loop antigenic site (VP1 residues 138 to 150) abolished infectivity of virus in suckling mice [37]. After ZB strain attenuation, the influence of the S142-R change in the −1 position and S147-A change in the +2 position of RGD is not clear (Figure 3(a)). The amino acid substitutions around the RGD motif suggest advantages of these substitutions in host cell recognition and binding during the attenuation process of the ZB strains. It is conceivable that these amino acid substitutions may modify the surface properties of the virion in a way that may reduce its virulence in cattle. The amino acids immediately following the RGD motif have a major influence on the ability of the different integrin species, and RGD +1 position is important in the receptor recognition process [38]. Residue phenylalanine at RGD +1 position of the cell-adapted rabbit-attenuated ZB/CHA/58(att) was different in other ZB strains, suggesting that this substitution may be associated with the cell adaptation in BHK-21 cells.

In the FMDV genomic P2 region, during the virulent ZB strain attenuation process, three common residues with a similar nature (N19-S, A74-T, and V136-I) of substitutions were found in 2B (Table 3), making their influence in attenuation difficult to assess. FMDV 2B protein is an integral membrane protein and localizes to endoplasmic reticulum-derived outer surface vesicles which are sites of genome replication [39]. In the 2C protein, four common amino acid substitutions were observed after attenuation (Table 3), and we suspect that amino acid substitutions K64-E and Y313-H play a role in viral virulence by interactions with several host factors during infection. FMDV 2C, with ATPase activity, localizes to membrane-associated virus-replicating complexes [39]. The 2C protein in the hepatitis A virus is one of the virulence genes in this virus [40]. In the case of the FMDV C-S8cl strain, residue T248-I mutation in 2C was not required for virulence of virus adaptation to infect the guinea pig [41], and residue R55-W mutation in 2C had regained competence in plaque development on BHK21 [42].

In the 3A protein, sequence analysis showed that no deletion or insertion mutations existed between the attenuated ZB strains and their parental viruses, while five common amino acid mutations were observed (Table 3). A residue substitution (A51-G) was only found in ZB/CHA/58(att), and another substitution (D107-N) was found in ZBRF188 and ZB/CHA/58(att) (Figure 3(b)). We propose that these amino acid substitutions are likely to be the major determinants of attenuation, especially residues E78-G, H80-C, K84-N, and R127-I, which will result in a less charged and more hydrophobic 3A protein than the one in virulent ZBCF22. Residue E78-G substitution was also observed in the FMDV R strain and its chick-attenuated R304 strain [43]. It was previously demonstrated that the Q44-R amino acid substitution in 3A of FMDV strain C-S8cl can mediate adaptation of FMDV to the guinea pig [41]. The 3A protein has been found to be associated with bovine attenuation of egg-adapted FMDV [44], and deletions in 3A have been shown to be associated with FMDV attenuation in cattle and high virulence in pigs [45, 46]. It was recently shown that the partial deletion in 3A can attenuate FMDV in cattle [47]. It would be interesting to examine by reverse genetics if these amino acid substitutions in the 3A protein of the ZB strain may contribute to an attenuation phenotype in cattle.

In conclusion, we identified genomic changes between the virulent and attenuated FMDV ZB strains in the untranslated regions, as well as in the protein coding regions, and discussed those changes correlated with the virus attenuation in context of the current knowledge of the FMDV's molecular biology. Study data indicated that candidate amino acid substitutions might play roles in the attenuation phenotype of the ZB strains. However, determining which and how those amino acid substitutions are directly involved in the attenuation of the virulent viruses will require animal experiments and cell infections to determine the pathogenesis of different mutated viruses. We are now using reverse genetic approaches to construct a series of mutants to confirm the virulent determinants during the attenuation process.

## Figures and Tables

**Table 1:** Primers used for amplification of the FMDV ZB strain genome.

Primer	Sequence (5′-3′)	Fragment	Coordinates^a^
F1	TTGAAAGGGGGCGCTAGGTCT	F1	1–22
R1011	CCTATTCAGGCGTAGAAGCTT	F1	991–1011
F916	CACTGGTGACAGGCTAAGGATG	F2	887–908
R2383	CCGTCATGTTGGTGCGTGGGTT	F2	2363–2384
F1608	ACGATCAGGAACCACTCAACG	F3	1608–1628
R4100	AGCTTGTACCAGGGTTTGGC	F3	4081–4100
F3970	CAGATGCAGGAGGACATGTC	F4	3970–3989
R6434	AGAGGCCAGGCATGGTGTC	F4	6406–6424
F5404	GAAAGGCCAACACGAAGCAGC	F5	5373–5393
R6944	TCCATGGCGTCAAGTCCGTCGACGC	F5	6920–6944
F6610	GGGTTGATCGTTGACACCAGAGA	F6	6610–6632
RES-dT_18_	GCGGCCGCGATATCT_18_	F6	Poly A

^a^Nucleotides position corresponds to the nucleotide sequence of the ZB/CHA/58(att) (GenBank number DQ533483).

**Table 2:** Nucleotide mutations of ZBCF22 and their attenuated strains in untranslated regions (UTRs)^†^.

Region	Position∗	ZBCF22	ZBRF168	ZBRF188	ZB/CHA/58(att)	YNBS/58	Corresponding residue in ref.
5′-UTR	573	C	G	G	G	C	G
3′-UTR	24	G	A	A	G	G	G

^†^Excluding poly(C) tract in the 5′-UTR fragment.

∗Nucleotides position corresponds to the nucleotide sequence of the ZB/CHA/58(att).

**Table 3:** Comparison of amino acid differences in the protein coding region between the virulent and attenuated ZB strains.

Region	Position∗	ZBCF22	ZBRF168	ZBRF188	ZB/CHA/58(att)	YNBS/58	Corresponding residue in ref.^†^
L^pro^	2	N	D	D	D	N	N
	143	M	L	L	L	M	M
	147	E	G	G	G	E	E

VP2	133	D	D	D	G	D	D
	184	V	L	L	L	V	V

VP1	4	A	T	T	T	A	A/T
	21	A	E	E	E	A	E
	45	P	L	L	L	P	P
	81	A	V	V	V	A	V
	142	S	R	R	R	S	M/R
	146	P	P	P	F	P	R/L/M
	147	S	A	A	A	S	A
	155	G	G	G	R	G	K/N/G/A

2A	8	K	K	K	E	K	K

2B	19	N	S	S	S	N	N
	74	A	T	T	T	A	A
	136	V	I	I	I	V	V

2C	6	V	I	I	I	V	I
	64	K	E	E	E	K	K
	135	T	T	I	I	T	T
	248	I	T	T	T	T	I/L
	313	Y	H	H	H	Y	H

3A	51	A	A	A	G	A	A
	78	E	G	G	G	E	E
	80	H	C	C	C	H	R
	84	K	N	N	N	K	K/Q
	97	A	P	P	P	A	A
	107	D	D	N	N	D	D
	127	R	I	I	I	R	R

3C^pro^	74	V	I	I	I	V	I

3D^pol^	84	R	H	H	H	R	R
	158	V	A	A	A	V	A/V
	378	H	Q	Q	Q	H	H

^*^The amino acids listed are at equivalent positions of ZB/CHA/58(att) (GenBank number DQ533483).

^†^The FMDV serotype Asia1 reference strains are YNBS/58(AY390432), PAK/54(AY593795), IND63/72(304994), IND 321/01(AY687333), IND 491/97(AY687334), ISRL13/63(AY593796), Asia1/3 Kimorn(AY593797), and Leb 3/83(AY593798).

**Table 4:** Synonymous nucleotides substitutions in the protein coding region between the virulent and attenuated ZB strains.

Region	Position∗	Virulent strains^†^	Attenuated strains^†^
L^pro^	1339	GC**A**	GC**G**
	1421	**C**TG	**T**TG
	1630	GT**G**	GT**A**

VP4	1807	CA**G**	CA**A**

VP2	2074	GA**C**	GA**T**
	2263	GG**C**	GG**G**
	2575	GT**A**	GT**G**

VP3	2980	GT**G**	GT**A**
	3019	GT**G**	GT**A**
	3076	TC**T**	TC**C**
	3169	AT**T**	AT**C**

VP1	3691	CG**T**	CG**C**

2B	4132	CT**C**	CT**T**

2C	4684	GC**T**	GC**C**
	4918	TT**C**	TT**T**
	5029	CT**A**	CT**G**

3A	5377	CA**G**	CA**A**

3B	6085	CC**T**	CC**C**
	6346	GA**C**	GA**T**

3D^pol^	7006	GA**G**	GA**A**
	7417	TG**C**	TG**T**

^*^The nucleotides listed are at equivalent positions of ZB/CHA/58(att) (GenBank number DQ533483). Nucleotide changes are indicated in bold.

^†^The virulent FMDV ZB strains included ZBCF22 and YNBS/58, and the attenuated strains included ZBRF168, ZBRF188, and ZB/CHA/58(att).
